# 12-HHT is associated with epithelial barrier enhancement and reduced inflammatory responses in colon organoids of normoganglionosis in Hirschsprung’s disease

**DOI:** 10.1371/journal.pone.0344140

**Published:** 2026-07-16

**Authors:** Kazuto Suda, Kumpei Abe, Yurina Nishimura, Masafumi Tanaka, Yuki Nagasako, Xuxuan Rao, Jianqin Zhang, Siyi Zeng, Kentaro Fujiwara, Shunsuke Yamada, Junya Ishii, Shiho Yoshida, Soichi Shibuya, Go Miyano

**Affiliations:** Department of Pediatric General and Urogenital Surgery, Juntendo University School of Medicine, Tokyo, Japan; Eötvös Loránd Research Network Biological Research Centre, HUNGARY

## Abstract

**Purpose:**

Hirschsprung-associated enterocolitis remains a major postoperative complication of Hirschsprung’s disease (HD), and impaired epithelial barrier integrity has been proposed as a contributing factor. In this study, we investigated whether 12-hydroxyheptadecatrienoic acid (12-HHT), an endogenous leukotriene B4 receptor 2 (BLT2) agonist, is associated with epithelial barrier enhancement and reduced inflammatory responses in patient-derived colonic organoids.

**Methods:**

Normoganglionic specimens from rectal/rectosigmoid HD at pull-through (HD-N; n = 8) and transverse colon specimens from anorectal malformation (ARM) at colostomy closure (n = 10) were used to generate colonic organoids. Epithelia were isolated using ethylenediaminetetraacetic acid and subsequently embedded in Matrigel. Baseline expression of *TJP1*, *TJP2*, *F11R* (encoding junctional adhesion molecule-A), *JAM2*, *CLDN1*, *CLDN3*, *CLDN4*) and *LTB4R2* (encoding BLT2) was assessed by qPCR and immunoblotting. Organoids were then treated with 12-HHT (0.4, 2, or 10 μM) for 7 days, followed by qPCR. Additional experiments assessed cytokine expression (*IL1B*, *IL6*) and TJPs after 24 h with tumor necrosis factor-α (TNF-α, 100 ng/mL) plus phosphate buffered saline or 12-HHT. Barrier function was evaluated using FITC–dextran influx assays.

**Results:**

HD-N and ARM organoids exhibited similar growth efficiencies. Baseline expression for *F11R*, *JAM2*, *CLDN1*, *CLDN3*, *CLDN4*, and *LTB4R2* was significantly lower in HD-N than in ARM. TJPs were upregulated by 12-HHT at 2 and 10 μM in both groups, with stronger effects in ARM. In HD-N organoids, 10 μM 12-HHT suppressed TNF-α-induced *IL1B* and *IL6* elevation mitigated tight junction proteins (TJPs) downregulation more effectively than 2 μM. 12-HHT attenuated TNF-α–induced FITC–dextran influx in HD-N organoids.

**Conclusion:**

12-HHT is associated with epithelial barrier enhancement and reduced inflammatory responses in HD-N organoids.

## Introduction

Hirschsprung’s disease (HD) is a congenital disorder characterized by functional bowel obstruction due to the absence of enteric ganglion cells in the rectum and distal colon [1–4]. The standard surgical treatment is resection of the aganglionic segment followed by pull-through of the proximal normoganglionic bowel [5,6]. Despite successful surgery, Hirschsprung-associated enterocolitis (HAEC) can occur even in the absence of obstructive symptoms and may be life threatening, with a reported incidence as high as 33% [7]. The risk of HAEC increases with the length of the aganglionic segment, particularly in cases of long segments, total colonic, or extensive aganglionosis [8,9]. Emerging evidence suggests that immature intestinal barrier function is not limited to the aganglionic segment but may also be present in the normoganglionic colon of patients with HD [10].

We previously reported that the normoganglionic colons of patients with HD showed significantly reduced expression of tight junction proteins (TJPs), particularly Claudin-4, along with decreased levels of its upstream regulator, Leukotriene B4 receptor 2 (BLT2) [8]. BLT2 was originally identified as a low-affinity receptor for Leukotriene B4, which enhances epithelial barrier function by facilitating Claudin-4 transcription in several tissues [11]. These alterations suggest that even the ganglionated segment has an intrinsic epithelial barrier vulnerability, which may predispose patients to bacterial translocation and mucosal inflammation. Accordingly, reduced Claudin-4 and BLT2 expression may be associated with the biological susceptibility of postoperative HAEC.

Eicosanoids derived from arachidonic acid metabolism are important regulators of intestinal epithelial homeostasis and barrier function. Previous studies have demonstrated that eicosanoid signaling contributes to the maintenance of epithelial integrity, tight junction regulation, and mucosal restitution in the intestine [12–14]. Therefore, eicosanoid-related signaling has attracted increasing interest as a potential therapeutic target for intestinal barrier dysfunction. 12-Hydroxyheptadecatrienoic acid (12-HHT), an endogenous ligand of BLT2, is produced from prostaglandin H_2_ during cyclooxygenase-mediated arachidonic acid metabolism through both thromboxane A synthase-dependent and -independent pathways [15]. Functionally, 12-HHT upregulates TJPs such as Claudin-4 and contributes to epithelial barrier maintenance. A recent study demonstrated that 12-HHT improves barrier function in an intestinal epithelial cell line, Caco-2 through BLT2 signaling [16]. However, its effects on epithelial barrier function in the context of HD, particularly using patient-derived intestinal organoid models, have not been investigated.

Organoid culture systems recapitulate the structural and functional properties of native tissues and have been widely recognized as valuable platforms in regenerative medicine and stem cell biology [17,18]. Recent reports have utilized gut-derived organoids to evaluate epithelial barrier integrity in HD, offering a promising platform for disease modeling [19,20]. Based on these insights, we hypothesized that 12-HHT may improve TJPs expression and barrier function in epithelial organoids derived from the normoganglionic colon in patients with HD. This study aimed to investigate the barrier-enhancing and potential anti-inflammatory effects of 12-HHT using patient-derived organoid cultures to propose a novel therapeutic approach to reduce the risk of HAEC following pull-through surgery.

## Materials and methods

### Study design

**Fig 1:**
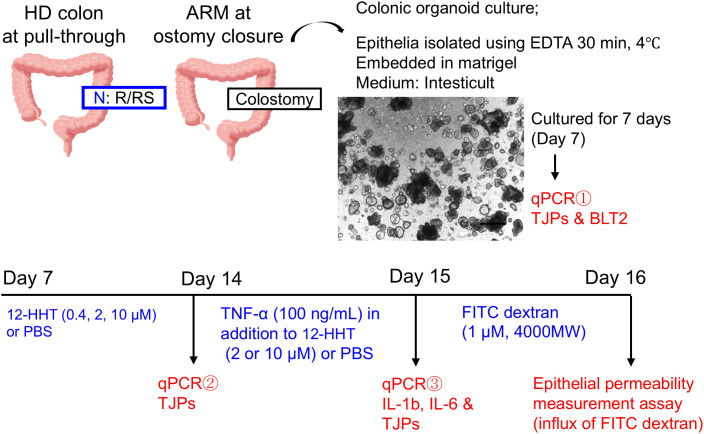
Study design. Epithelial cells were isolated using EDTA treatment from colonic specimens obtained from patients with HD at pull-through surgery and from patients with ARM at the time of ostomy closure. Organoids were cultured for 7 days, followed by baseline qPCR analysis of TJPs and BLT2. Organoids were passaged and treated with 12-HHT from days 7 to 14, followed by qPCR analysis of the TJPs. Using the same organoid line, organoids were subsequently stimulated with TNF-α in combination with 12-HHT for 24 h, and qPCR for *IL1β*, *IL6*, and TJPs was performed on day 15. Epithelial permeability was evaluated on day 16 using an FITC–dextran influx assay. R/RS; rectal or rectosigmoid colon, N; normoganglionosis.

Patients were recruited between 3 June 2024 and 27 November 2025. The subjects were colonic specimens of the normoganglionic segment from the rectal or rectosigmoid type of HD at pull-through surgery (HD-N) and transverse colon specimens from anorectal malformations (ARM) at colostomy closure, which served as non-HD controls (Fig 1). Human colonic epithelial organoids were cultured for seven days and subjected to experimental paradigms.

First, the baseline expression of TJPs and BLT2 was assessed by qPCR on day 7. In the second paradigm, organoids were passaged and then treated with different concentrations of 12-HHT (0.4, 2, or 10 μM) or phosphate buffered saline (PBS) from day 7 to day 14. TJPs expression was evaluated using qPCR on day 14. Using the same organoid line, organoids were subsequently stimulated with TNF-α (100 ng/mL) in the presence of 12-HHT (2 or 10 μM) or PBS for 24 h, and qPCR for *IL1β*, *IL6*, and TJPs was performed on day 15. Epithelial permeability, assessed in the same organoid line, was measured on day 16 using FITC–dextran influx assay.

### Colonic organoid culture

The human colonic organoids culture was established using a modified protocol based on previously published methods [21,22]. Briefly, the muscular layer of the resected colon was manually removed using scissors and the remaining tissue was minced and gently rinsed with Dulbecco’s PBS (D-PBS) until the supernatant became clear. After discarding D-PBS, the minced tissue was incubated in 5 mM of Ethylenediaminetetraacetic acid (EDTA) for 30 minutes at 4°C with gentle agitation. Following manual vigorous pipetting, the resulting suspension was passed through a 100-µm cell strainer and centrifuged at 100 × g for 3 minutes at 4°C to isolate epithelial cells. The cell pellet was resuspended and embedded in a 25-µL droplet of Matrigel (Corning, USA), which was placed at the center of each well of a pre-warmed 24-well plate. Organoids were maintained in IntestiCult Organoid Growth Medium (Human) (StemCell Technologies, Canada), which was replaced every three days. For passaging, organoids were harvested from the Matrigel using Cell Recovery Solution (Corning, USA), resuspended in fresh Matrigel, and subsequently replated and cultured in growth medium.

### Reagents treatments (12-HHT and TNF-α)

12-HHT (H-1640, Sigma-Aldrich), a commercially available synthetic compound, was used as a pharmacological activator of BLT2 signaling. It was prepared as 100 µM aliquots in PBS after complete evaporation of ethanol and used at final working concentrations of 0.4, 2.0, and 10.0 µM in the organoid culture medium. TNF-α was prepared as 10 µg/mL aliquots in PBS, following previously published methods, and applied at a final working concentration of 100 ng/mL [23].

### Quantitative PCR (qPCR)

Total RNA was extracted from the organoid cells using an RNeasy Micro Kit (QIAGEN, Germany). qPCR was performed for TJP-related genes (*TJP1*, *TJP2*, *F11R*, *JAM2*, *CLDN1*, *CLDN3*, *CLDN4*) [24–27], epithelial differentiation and maturation markers, including goblet cell–associated markers (*KLF4*, *TFF3*, and *MUC2*) [28,29], and pro-inflammatory cytokines (*IL1B* and *IL6*) [30], according to the manufacturer’s specifications. All results were normalized to *GAPDH* conventionally. The primer sequences used for qPCR are listed in Table 1.

**Table 1:** Primer sequences used for qPCR.

Gene	Forward Primer (5′ → 3′)	Reverse Primer (5′ → 3′)
*TJP1*	ACCAGTAAGTCGTCCTGATCC	TCGGCCAAATCTTCTCACTCC
*TJP2*	GGGAAGGTCGCTGCTATTGT	CTCTCGCTGTAGCCACTCC
*F11R*	GTGCCTACTCGGGCTTTTCTT	GTCACCCGGTCCTCATAGGAA
*JAM2*	TTGTGAAGTTAGTGCCCCATC	CCCTTCTTTGTCTTGACATCGTA
*CLDN1*	CCTCCTGGGAGTGATAGCAAT	GGCAACTAAAATAGCCAGACCT
*CLDN3*	AACACCATTATCCGGGACTTCT	GCGGAGTAGACGACCTTGG
*CLDN4*	TGGGGCTACAGGTAATGGG	GGTCTGCGAGGTGACAATGTT
*LTB4R2*	AGAAGGATGTCGGTCTGCTAC	CCAAGCTCCACACCACGAA
*KLF4*	CGGACATCAACGACGTGAG	GACGCCTTCAGCACGAACT
*TFF3*	CCAAGCAAACAATCCAGAGCA	GCTCAGGACTCGCTTCATGG
*MUC2*	GGAGATCACCAATGACTGCGA	GAATCGTTGTGGTCACCCTTG
*IL1B*	AGCTACGAATCTCCGACCAC	CGTTATCCCATGTGTCGAAGAA
*IL6*	ACTCACCTCTTCAGAACGAATTG	CCATCTTTGGAAGGTTCAGGTTG
*GAPDH*	GGAGCGAGATCCCTCCAAAAT	GGCTGTTGTCATACTTCTCATGG

### Immunoblotting

Proteins were extracted from organoid cells using RIPA buffer supplemented with a protease inhibitor cocktail (1:50, Roche) to perform immunoblotting according to a previously published technique [8] and the manufacturer’s instructions using the following primary antibodies: CLDN4 (1:5000; Invitrogen, Cat# 32-9400, clone 3E2C1) and anti-GAPDH (14C10, 1:5000; Cell Signaling Technology). Signals detected using horseradish peroxidase–conjugated secondary anti-rabbit (1:10,000, Jackson ImmunoResearch) or anti-mouse antibody (1:10,000, Jackson ImmunoResearch) were visualized using Image Quant LAS 4000 (GE Healthcare).

### Quantification of organoid size and number

For the quantitative analysis of organoid growth, both organoid size and number were assessed using ImageJ software. For each experimental condition, including comparisons between HD-N and ARM organoids as well as TNF-α treatment with or without 12-HHT, five representative images were obtained. All organoids within each image were analyzed, and the projected area of each organoid was measured (organoids with a long-axis diameter >100 μm were included), while the total number of organoids per image was also counted. The mean organoid area and number for each condition were calculated and normalized to the control group, which was set to a mean value of 1. Data are presented as relative ratios.

### Measurement of FITC

FITC–dextran (MW 4000) (MedChemExpress, HY-128868A) was prepared as 1 mM aliquots in dimethyl sulfoxide (DMSO). The permeability of organoid lumens to fluorescent markers was tested using FITC–dextran, according to previously reported methods [31]. Organoids were washed twice with PBS and incubated with FITC–dextran at a final concentration of 1 µM in organoid culture medium for 6 h at room temperature. The marker solution was removed and the organoids were washed five times with PBS. Fluorescence intensity of FITC remaining in the organoid lumen was captured using a BZ-X700 fluorescence microscope (Keyence, Japan) with a GFP filter and quantified using region of interest (ROI) analysis in ImageJ for each image. The mean values were calculated from five images per treatment. Intensities were normalized by subtracting the fluorescence of the PBS + DMSO group (negative control, without TNF-α), and the corrected values were expressed as a ratio relative to the PBS + TNF-α group for graphing.

### Trypan blue assay

Number of viable cells was assessed using a trypan blue exclusion assay, as previously described [32]. Briefly, cells were trypsinized with TrypLE™ Express Enzyme (Thermo Fisher Scientific, USA; Cat# 12605-010) at 37°C for 5 minutes. The enzymatic reaction was stopped by resuspending the cells in 1 mL of PBS (Nacalai Tesque Inc., Japan; Cat# 27575-31) supplemented with 1% fetal bovine serum (FBS; Thermo Fisher Scientific, USA; Cat# 10270-106). Cells stained with 1 mL of 0.5% trypan blue solution (Nacalai Tesque Inc., Japan; Cat# 29853-34) were immediately loaded onto a hemocytometer (Cat #480200; Corning, USA) and the number of unstained viable cells within a 1 × 1 mm grid was counted under a stereomicroscope.

### Statistics

All data are expressed as mean value ± standard deviations, except for age at specimen collection, which is presented as median (range). Differences between the two groups were tested for statistical significance using an unpaired t-test. All statistical tests were two-sided; *p* value of 0.05 or less was considered statistically significant.

### Ethics

This study was approved by the Institutional Review Board committee of Juntendo University School of Medicine (Institutional Review Board number: E21-0173) and complied with the Helsinki Declaration (2008). Written informed consent was obtained from the parents or legal guardians of all participating minors prior to specimen collection.

## Results

### Patient’s characteristics

Colonic samples were obtained from eight patients with HD and 10 patients with ARM, as summarized in Table 2. The median (range) age at specimen collection differed between groups (HD 2.67 (0.25–10.0) years; ARM 0.71 (0.50–1.83) years) (*p* < 0.05). Samples from patients with HD were primarily obtained from the sigmoid colon, whereas samples from patients with ARMs were derived exclusively from the transverse colon.

**Table 2:** Patients’ demographic.

	HD-N (n = 8)	ARM (n = 10)	*p* value
Age at specimen collection (year)	2.67 (0.25–10.0)	0.71 (0.50–1.83)	<0.05
Female:Male	4:4	5:5	N. S
Region for sample collection			<0.0001
Transverse colon	0	10 (100%)	
Descending colon	1 (12.5%)	0	
Sigmoid colon	7 (87.5%)	0	

HD-N: normoganglionic specimens from Hirschsprung’s disease,

ARM: Anorectal malformation, N.S: not significant.

### Reduced baseline expression of tight junction proteins and BLT2 in HD-derived organoids

**Fig 2:**
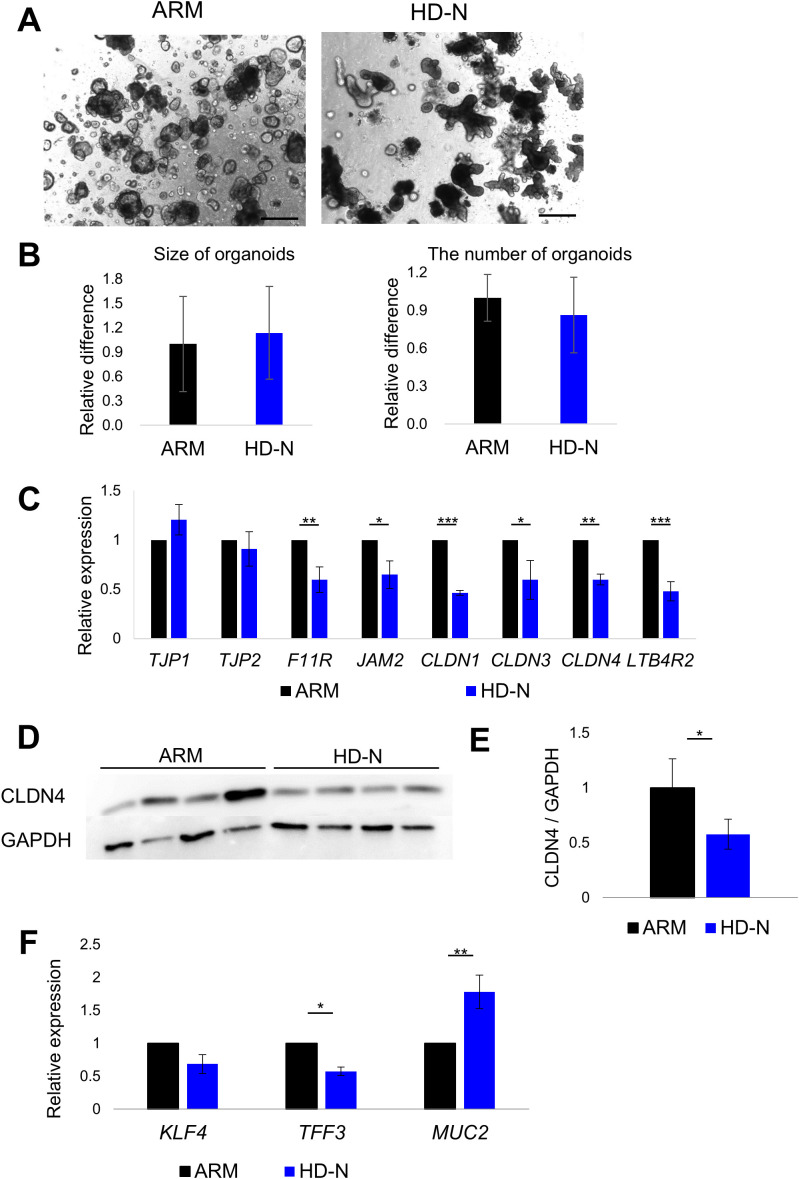
Baseline expression of tight junction–related genes and BLT2 in HD-N and ARM organoids. **A)** Representative images of ARM and HD-N organoids formation on days 7. Scale bar: 500 μm. **B)** Quantitative analysis of organoid size and number based on ImageJ measurements using multiple independent images. No significant differences were observed between HD-N and ARM organoids. **C)** Relative expression levels of TJPs and *LTB4R2* (encoding BLT2) in HD-N and ARM organoids graphed by qPCR. *p < 0.05, **p < 0.01, ***p < 0.001. **D)** Representative immunoblotting images showing Claudin-4 protein levels in ARM and HD-N organoids. **E)** Quantitative analysis of the CLDN4/GAPDH ratio based on immunoblotting. *p* < 0.05. **F)** Relative expression levels of epithelial differentiation markers in HD-N and ARM organoids graphed by qPCR. *p < 0.05, **p < 0.01.

Organoids derived from HD-N and ARM samples exhibited comparable growth efficiencies (Fig 2A). Quantitative analysis of those organoid size and number using multiple independent images revealed no significant differences between HD-N and ARM organoids (Fig 2B). Baseline analysis of day-7 organoids demonstrated that HD-N-derived organoids showed significantly lower expression of multiple TJP–associated genes than ARM controls, including *F11R*, *JAM2*, *CLDN1*, *CLDN3*, and *CLDN4* (Fig 2C). Expression of *LTB4R2* (BLT2) was also markedly low in HD-derived organoids (Fig 2C). Immunoblotting demonstrated lower Claudin-4 protein levels in HD-N organoids than in ARM organoids (*p* < 0.05) (Fig 2D, 2E). Among the differentiation markers, qPCR showed that the expression levels of *TFF3* (*p* < 0.05) and *KLF4* were lower in HD-N organoids than those in ARM organoids, whereas *MUC2* expression was higher in HD-N organoids (*p* < 0.01) (Fig 2F).

### 12-HHT modulates not only TJP expression but also differentiation on colonic organoids culture

**Fig 3:**
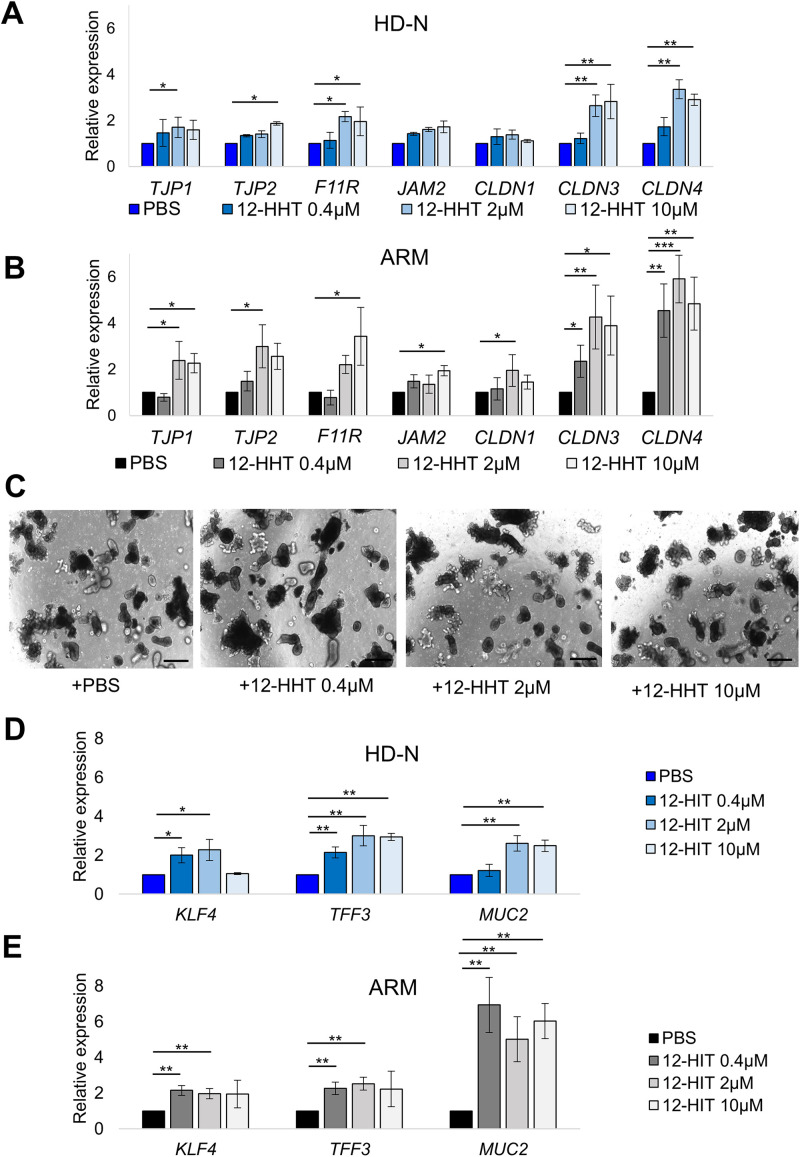
Effects of 12-HHT on barrier-related gene expression in HD-N and ARM organoids. **A)** Concentration-dependent changes in TJP gene expression in HD-N organoids treated with 12-HHT (0.4, 2, or 10 µM) for 7 days (day 7–14). *p < 0.05, **p < 0.01, ***p < 0.001. **B)** Concentration-dependent changes in TJP gene expression in ARM organoids treated with 12-HHT (0.4, 2, or 10 µM) for 7 days (day 7–14). *p < 0.05, **p < 0.01. **C)** Organoids morphology treated with 12-HHT (0.4, 2, or 10 µM) for 7 days (day 7–14). Scale bar: 500 μm. **D)** Expression of epithelial differentiation markers in HD-N organoids treated with 12-HHT (0.4, 2, or 10 µM) for 7 days (day 7–14). *p < 0.05, **p < 0.01. **E)** Expression of epithelial differentiation markers in ARM organoids treated with 12-HHT (0.4, 2, or 10 µM) for 7 days (day 7–14). **p < 0.01.

Treatment with the BLT2 agonist 12-HHT from day 7 to day 14 resulted in a concentration-dependent upregulation of several TJP genes in HD-N organoids, with significant increases observed particularly for *CLDN3* and *CLDN4* at 2 µM and 10 µM (Fig 3A). This upregulation was more pronounced in ARM organoids (Fig 3B). No significant morphological changes were observed in the HD-N organoids after 12-HHT treatment (Fig 3C). The mRNA expression levels of *TFF3*, *KLF4*, and *MUC2* were upregulated by 12-HHT treatment in both the ARM and HD-N groups, with a more pronounced increase observed in the ARM group (Fig 3D, 3E).

### Effects of 12-HHT on cytokine responses on colonic organoids culture

**Fig 4:**
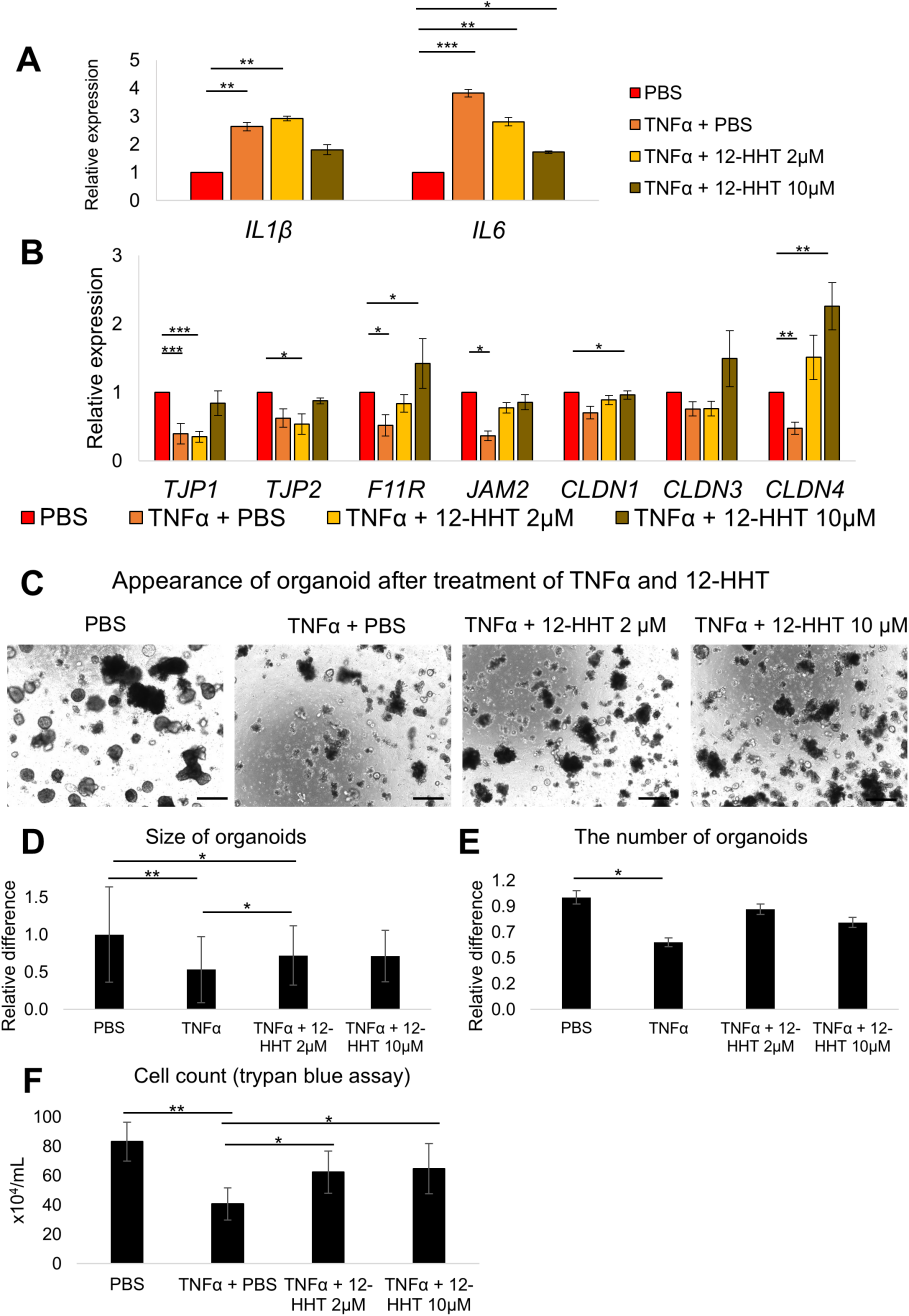
Protective effects of 12-HHT against TNF-α–induced inflammatory signaling and barrier dysfunction in HD-N organoids. **A)** Relative expression of inflammatory cytokines after 24-h stimulation with TNF-α (100 ng/mL) with or without 12-HHT (2 or 10 µM). * *p* < 0.05, **p < 0.01, ***p < 0.001. **B)** Relative expression of tight junction genes after 24-h stimulation with TNF-α (100 ng/mL) with or without 12-HHT (2 or 10 µM). *p < 0.05, **p < 0.01, ***p < 0.001. **C)** Representative images of organoid morphology after 24-h stimulation with TNF-α (100 ng/mL) with or without 12-HHT (2 or 10 µM). Scale bar: 500 μm. **D)** Quantitative analysis of organoid size based on ImageJ measurements. *p < 0.05, **p < 0.01. **E)** Quantitative analysis of organoid number based on ImageJ measurements. *p < 0.05. **F)** Quantitative analysis of cell counts determined by the trypan blue exclusion assay. *p < 0.05, **p < 0.01.

Exposure of organoids to TNF-α for 24 h induced robust upregulation of inflammatory cytokines *IL1β* and *IL6* and reduced expression of multiple TJP genes (Fig 4A, 4B). Co-treatment with 12-HHT significantly suppressed TNF-α–induced cytokine expression (Fig 4A) and restored TJP expression in a concentration-dependent manner (Fig 4B). Representative morphological images and quantitative analysis showed that TNF-α treatment, reduced both mean area and the number of organoids, whereas 12-HHT treatment ameliorated these reductions (Fig 4C, 4D, 4E). Consistent with the morphological data, trypan blue assays demonstrated that TNF-α stimulation decreased viable cell counts, an effect that was reversed by co-treatment with 12-HHT (Fig 4F).

### 12-HHT improves permeability of colonic organoids

**Fig 5:**
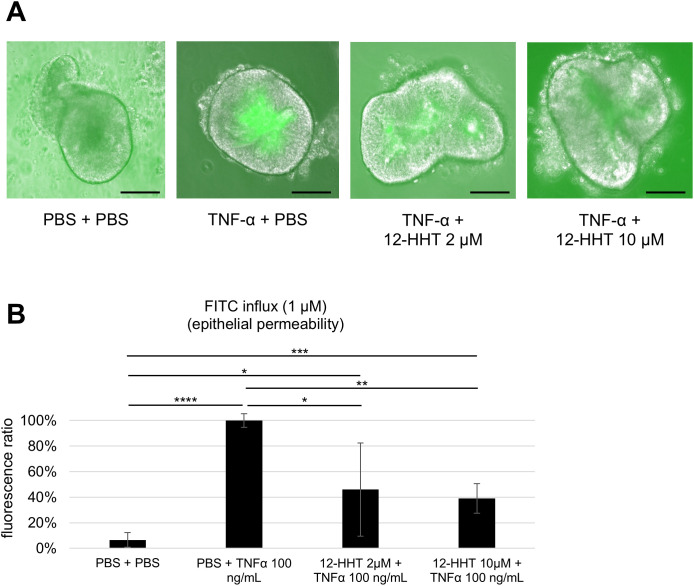
Reduction of TNF-α–induced epithelial permeability by 12-HHT in HD-N organoids. **(A)** Representative fluorescence images of FITC–dextran (1 µM) influx after 24-h exposure to TNF-α with or without 12-HHT in HD-N organoids. Scale bar: 100 μm. **(B)** Quantitative analysis of FITC–dextran influx in HD-N organoid based on ImageJ measurements. *p < 0.05, **p < 0.01, ***p < 0.001, ****p < 0.0001.

FITC–dextran influx assays demonstrated markedly increased epithelial permeability in HD-derived organoids by TNF-α (Fig 5A). Co-treatment with 12-HHT significantly reduced FITC influx, with the strongest effect observed at 10 µM (Fig 5A, 5B). These findings indicate that BLT2 activation mitigates TNF-α–induced impairment of epithelial barrier.

## Discussion

This study demonstrated that colonic organoids from the normoganglionic segment of patients with HD exhibit intrinsic epithelial barrier impairment, characterized by reduced expression of multiple TJPs and their upstream regulator, BLT2. Importantly, the activation of BLT2 by its endogenous ligand 12-HHT upregulated TJPs expression, reduced pro-inflammatory cytokine induction, and retained epithelial permeability even under inflammatory stress. These findings extend our previous observations in patient tissues and provide functional evidence that epithelial vulnerability in HD is not restricted to the aganglionic segment, but persists in the normoganglionic bowel [8]. Collectively, our results suggest that impaired epithelial barrier regulation, potentially involving BLT2–related pathways, may contribute to HAEC susceptibility, and that these findings provide a basis for further investigation into epithelial barrier–targeted strategies. The novelty of the present study lies in demonstrating that epithelial barrier dysfunction in the normoganglionic colon can be improved under inflammatory stress through BLT2 activation, using human gut-derived organoids from patients with HD. Through the introduction of an organoid culture system, we experimentally validated this concept in a human context, which could not be directly examined in vivo.

Interestingly, the TJP-upregulating effect of 12-HHT was more pronounced in the ARM organoids than in HD-N. This is likely attributable to the low baseline expression of BLT2 in the HD-N organoids, which may allow for less efficient upregulation of TJPs. These findings suggest that the magnitude of the 12-HHT effect depends on the baseline level of BLT2 expression. Similarly, the upregulation of the differentiation markers *KLF4*, *TFF3*, and *MUC2* in response to 12-HHT was less pronounced in HD-N organoids than in ARM organoids. This attenuated response may be attributable to disease-specific epithelial vulnerability in the normoganglionic colon of patients with HD, whereas differences in baseline BLT2 expression levels may also contribute to the observed effects.

Previous studies reported findings consistent with our observations regarding the effects of BLT2 activation mediated by 12-HHT. In a dextran sodium sulfate–induced murine colitis model, BLT2-deficient mice exhibited more severe disease, characterized by greater body weight loss, enhanced intestinal inflammation, increased expression of pro-inflammatory cytokines, and augmented accumulation of activated macrophages [33]. Moreover, the 12-HHT–BLT2 axis enhances epithelial barrier function by upregulating Claudin-4 expression and promoting the recovery of transepithelial electrical resistance by activating the p38 MAPK pathway or the p38–PKC signaling axis [11,34]. Collectively, these studies support the protective role of BLT2 against inflammatory injury, whereby BLT2 signaling strengthens the integrity of the epithelial barrier in the intestine and other epithelial tissues, thereby conferring resistance to inflammatory insults.

Other endogenous ligands of BLT2, including 12-HHT, Hydroxyeicosatetraenoic acids (HETEs), and Hydroxyoctadecadienoic acids (HODEs), have been reported to regulate intestinal epithelial cell functions [35,36]. In particular, studies using human Caco-2 intestinal epithelial cells demonstrated that HETEs and HODEs promote epithelial cell proliferation and survival, potentially involving BLT2 as well as multiple signaling pathways such as ERK and p38 [35,36]. These findings suggest that BLT2-associated lipid mediators exert diverse biological effects on epithelial homeostasis. In addition, 12-HHT has been implicated in epithelial barrier regulation, including modulation of TJPs, possibly through BLT2-mediated signaling, as suggested by the present study together with previous reports. Recent studies have also suggested that other BLT2-associated lipid mediators, including resolvins, may promote epithelial repair and mucosal healing through BLT2-related pathways, thereby potentially contributing to restoration of intestinal barrier homeostasis [37]. Further comparative evaluation of these lipid mediators in epithelial barrier function would be of interest in future studies.

Furthermore, KLF4, a transcription factor that promotes the terminal differentiation of intestinal epithelial cells; TFF3, a goblet cell–derived secretory peptide involved in mucosal protection and epithelial restitution; and MUC2, a mucin produced by mature goblet cells [28,29], were transcriptionally activated by 12-HHT in both HD-N and ARM organoids. Previous studies have demonstrated that 12-HHT signaling promotes epithelial repair and cell migration [38,39]. However, its direct role in the regulation of epithelial differentiation and maturation remains unclear. Indeed, even in our experimental system, it is difficult to determine whether 12-HHT directly promotes epithelial differentiation, or whether its epithelial protective effects preserve differentiated cell populations, thereby resulting in the increased expression of differentiation-associated markers. Additionally, the baseline expression levels of BLT2 and Claudin-4 were lower in the HD-N organoids than in the ARM organoids. A subclinical inflammatory state has been reported in the intestinal epithelium of patients with HD, even without active enterocolitis [40,41]. Thus, reduced expression of differentiation markers such as *KLF4* and *TFF3* in HD-N organoids may reflect an underlying epithelial vulnerability associated with chronic, low-grade inflammatory stress rather than an active inflammation.

This study has some limitations. First, patients were not stratified by postoperative HAEC history because the cohort predominantly included short-segment HD cases, which have a relatively low HAEC incidence; none developed HAEC before pull-through surgery. Second, obtaining colonic specimens from healthy children is ethically challenging. In this context, ARM tissue has been widely used as a clinically realistic control because it is associated with fewer inflammatory background abnormalities than HD [8,41,42]. Although the anatomical location and age at specimen collection differed between the HD and ARM groups in the present study, previous reports, including ours, have provided limited evidence that these factors alone account for the marked differences observed in the expression of tight junction proteins such as Claudin-4 [8,43]. Moreover, the organoid model represents epithelial cells alone and does not incorporate immune cells, neuronal components, or intestinal microbiota, all of which are recognized modulators of HAEC pathophysiology. Accordingly, more complex systems, such as co-culture approaches or microfluidic organ-on-chip models, may be required to better elucidate the interactions among epithelial cells, immune cells, and microbiota in HD. In addition, the relatively weaker response to 12-HHT observed in HD-N organoids compared to ARM organoids warrant further investigation, including evaluation of higher ligand concentrations, longer treatment durations, and alternative strategies for BLT2 activation.

In conclusion, our findings suggest that BLT2–related signaling is associated with the improvement of epithelial barrier integrity in HD-derived tissues. These observations provide a potential mechanistic basis for epithelial barrier regulation, which may be relevant to HAEC susceptibility after pull-through surgery.

## Supporting information

S1 Data Original, uncropped blot images. The membranes were cut at approximately 50 kDa during the experimental procedure. Therefore, only proteins below this molecular weight are shown. The provided images represent the original, unadjusted membrane images retained at the time of data acquisition. In each image, two membrane sections are visible vertically, and the upper membrane corresponds to the data presented in the main figures. Full-length membrane images are not available, as only the membrane regions relevant to the target proteins (CLDN4 and GAPDH) were retained.
(PDF)

S2 Data Raw data underlying all quantitative analyses. Numerical values used for statistical analyses and graph generation.
(XLSX)
