# Supplementary figures and images for: 12-HHT is associated with epithelial barrier enhancement and reduced inflammatory responses in colon organoids of normoganglionosis in Hirschsprung’s disease
Source: PLoS One. 2026 Jul 16;21(7):e0344140. doi: 10.1371/journal.pone.0344140 (PMC13374890; doi:10.1371/journal.pone.0344140)

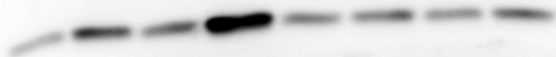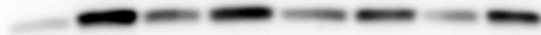

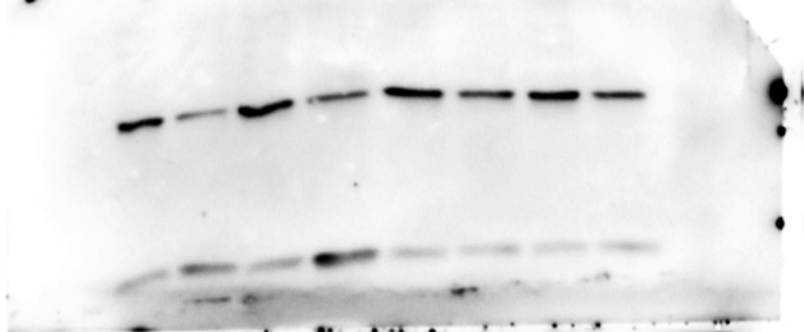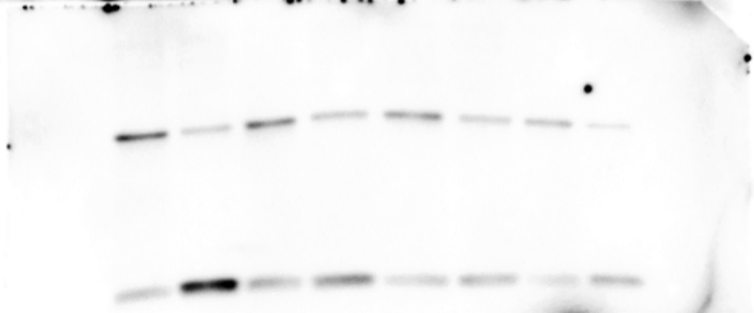

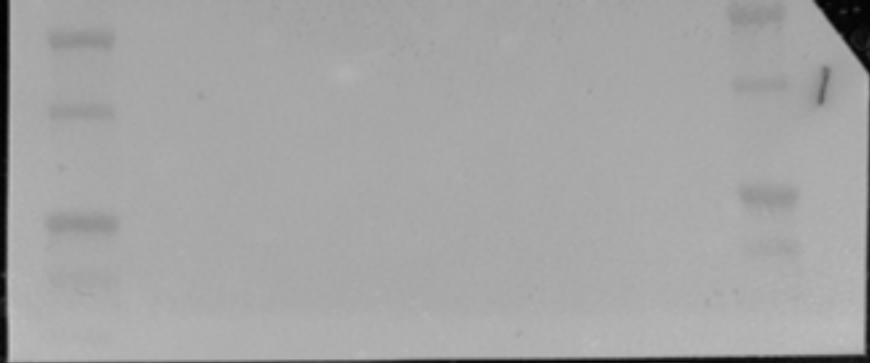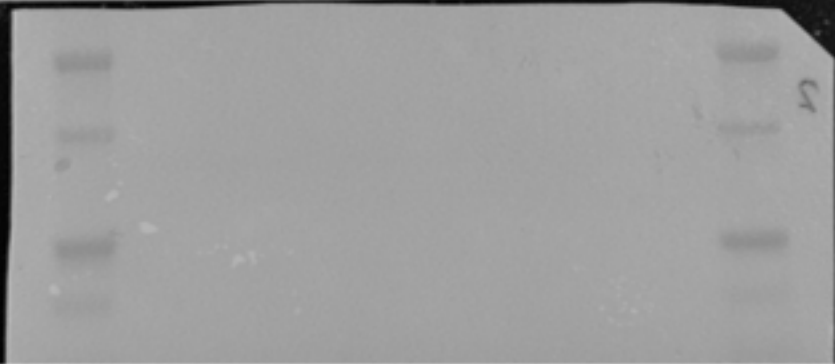

Supplement: S1 Data — The membranes were cut at approximately 50 kDa during the experimental procedure. Therefore, only proteins below this molecular weight are shown. The provided images represent the original, unadjusted membrane images retained at the time of data acquisition. In each image, two membrane sections are visible vertically, and the upper membrane corresponds to the data presented in the main figures. Full-length membrane images are not available, as only the membrane regions relevant to the target proteins (CLDN4 and GAPDH) were retained. (PDF) [file pone.0344140.s001.pdf]
